# Parental Well-being Surrounding First Birth as a Determinant of Further Parity Progression

**DOI:** 10.1007/s13524-015-0413-2

**Published:** 2015-08-04

**Authors:** Rachel Margolis, Mikko Myrskylä

**Affiliations:** Department of Sociology, University of Western Ontario, Social Science Center #5326, London, Ontario N6A 5C2 Canada; Laboratory of Fertility and Well-being, Max Planck Institute for Demographic Research, Konrad-Zuse-Str. 1, 18057 Rostock, Germany; Department of Social Policy, London School of Economics and Political Science, Houghton Street, London, WC2A 2AE UK; Population Research Unit, Department of Social Research, University of Helsinki, P.O. Box 18, 00014 Helsinki, Finland

**Keywords:** Low fertility, Parity progression, Subjective well-being, Transition to parenthood

## Abstract

A major component driving cross-country fertility differences in the developed world is differences in the probability of having additional children among those who have one. Why do people stop at having only one child? We hypothesize that the experience of the transition to parenthood is an important determinant of further fertility. Analyzing longitudinal data from Germany, we find that the experience during the transition to parenthood, as measured by changes in subjective well-being, predicts further parity progression. A drop in well-being surrounding first birth predicts a decreased likelihood of having another child. The association is particularly strong for older parents and those with higher education: these characteristics may be related to the ability or willingness to revise fertility plans based on prior experiences. Parents’ experience with the first birth is an important and understudied factor in determining completed family size, and policy-makers concerned about low fertility should pay attention to factors that influence the well-being of new parents.

## Introduction

Period fertility declined in much of the developed world to below-replacement levels in the late twentieth century. By the early twenty-first century, more than one-half of the world’s population lived in countries with below-replacement fertility (Wilson 2004). Despite recent increases in fertility (Goldstein et al. 2009; Myrskylä et al. 2009), low fertility continues to be one of the key social challenges for the developed world because of its implications for population aging (European Commission 2006).

Research has identified several important social and demographic determinants of low period fertility. The rise of individualistic values and the increase of women into the paid labor force have led to low levels of desired and actual fertility (Brewster and Rindfuss 2000; Lesthaeghe and van de Kaa 1986) and to fertility postponement. The postponement of births also suppresses period measures of fertility (Bongaarts and Feeney 1998). Although postponement is a major contributing factor to low period fertility (Myrskylä et al. 2013; Sobotka 2004), a decrease in quantum driven by stopping at one or two children, or having none, is also important (Frejka 2008).

Qualitative work suggests that the way in which new mothers and fathers experience becoming parents is an important determinant of further fertility plans (Newman 2008). However, no quantitative work has analyzed how the experience of becoming a parent influences future fertility. Newman’s (2008) qualitative results suggest that this represents an important gap in our understanding of fertility behavior. In this article, we examine how the experience of becoming a parent, as measured by changes in parental well-being during the process of having a first child, influences further parity progression.

We argue that the relationship between parents’ well-being and fertility at the first birth is important for individuals’ further reproductive behavior. Parents’ experiences with the transition to parenthood would be unlikely to be a major factor if people predicted well how they will experience parenthood. However, although parenthood itself is mostly expected, the experience of parenthood is often unexpected, in both positive and negative ways. For example, people underestimate the daily burden of caring for a child (Dyck 1990) but may not anticipate the positive and playful aspects of parenthood. Thus, the unexpected dimensions of parenthood may shape future fertility behavior up or down. If parenthood is more difficult than many new parents expect, and this suppresses parity progression, then micro-level patterns and individual experiences can have large effects on aggregate fertility.

### Parents’ Experience of the Transition to Parenthood

Fertility is a choice for most people in the developed world. Before people have children, they are uncertain about what pregnancy and childrearing are like. They may observe how peers or other family members cope with children, but they have no direct experience. After having a first child, though, they learn firsthand about parenting. Therefore, having a second child is a more informed decision than having a first. The experience of the transition to parenthood will inform new parents’ decisions about whether to have another child. If having a first child is an overall positive experience, or more positive than anticipated, then people should be more likely to have another. However, if the transition to parenthood is very difficult or more difficult than expected, then people may choose to remain at parity 1.

Parity progression as learning aligns with several theoretical frameworks, which all lead to similar hypotheses. Learning theories in psychology predict that people will avoid activities that they anticipate will negatively affect their physical or mental health (Rotter 1954). Similarly, the theory of planned behavior (Ajzen 1991; Ajzen and Klobas 2013) predicts that the experience with a first child influences the perceptions of the potential consequences of a second, which influences subsequent fertility behavior. Also, sequential decision theory (Wald 1947) and the conjunctural theory of action (Johnson-Hanks et al. 2011) posit that people make decisions in the present by taking into account their past experiences; a decision about having a baby today will depend on how a past decision has affected well-being.

Earlier demographic research, much of it qualitative, touched on the potential effect of subjective parenting experiences on further parity progression (Callan 1985; Cartwright 1976; Newman 2008; Presser 2001). For example, Presser (2001) predicted that new mothers would be shocked by the unrelenting demands of childrearing, which would discourage additional births. Recent qualitative work conducted among new parents in Australia explicitly examined how the experience of a first birth shapes fertility intentions and behavior (Newman 2008). “The balance of negative and positive experiences was important in decisions about how many times to ‘go through’ the Baby Stage again, although it was perhaps less influential on those who had had a strong desire over their lifetime for a particular family size” (Newman 2008:15). Surprisingly, positive experiences were the minority, but these positive experiences positively affected fertility. More common in Newman’s study was the parenting experience as a “parity progression hurdle” if the pregnancy, birth, or baby stage was particularly difficult or unexpectedly stressful.

Difficulties experienced by new parents that affected their achieved family size fell into three categories. First, new parents reported being strongly affected by difficulties conceiving and experiences of pregnancy. New mothers reported that their medical conditions, physical pain, and pregnancy nausea conflicted with their desire to work, and new fathers were concerned about medical issues for their partners (Newman 2008). Second, the experience of the birth influenced new parents’ desired family size. Long laboring or complications with Cesarean sections shaped parents’ desire not to “go through that again” (Newman 2008:7). Third and most importantly, two-thirds of Newman’s respondents reported that difficulties in the first year after a birth led to downward revisions of plans for additional children. The continuous and intense nature of childrearing in the first year was stressful for most parents, especially for those who had limited knowledge of baby care and social support. Parents with more than one child report that exhaustion in the baby stage was greatest with the first baby, especially if the exhaustion was unexpected. Other important factors for temporarily or permanently postponing having further children were trouble breast-feeding, sleep deprivation, depression, domestic isolation, and relationship breakdown. This qualitative research highlights the importance of the psychosocial experience of the transition to parenthood, but no quantitative work has tested this hypothesis or examined its relative importance net of other factors known to affect final parity.

### Subgroup Differences in the Importance of the Transition to Parenthood for Parity Progression

Parents’ experience with a first birth may be a more important factor in determining final parity for some groups rather than others. For example, parenting experiences may be more important for women than men in deciding whether to have another child because women physically experience the pregnancy and birth, do more childcare, and are more likely to take leave from work (Bianchi et al. 2000; Haas 2003). Moreover, the woman’s fertility preferences may carry more weight in the couple’s decision making than the man’s (Testa et al. 2011, 2014).

The transition to parenthood may be a more or less important predictor of further parity progression for parents of different age or different socioeconomic status (SES) groups, although the expected pattern is ambiguous. On the one hand, parenting experiences might be more critical in determining final parity for high-SES or older parents, perhaps because of a greater absolute opportunity cost of unpleasant physical consequences of pregnancy or parenting for high-SES working parents or those who are more advanced in their career (Morgan and Rackin 2010; Quesnel-Vallée and Morgan 2003). High-SES parents may also value their careers more and therefore may be more likely to stop after one child if they decide they cannot accomplish their professional goals after having another child (Newman 2008; Presser 2001). Men and women in higher-status occupations have higher average levels of work-to-home conflict than those in low-status occupations, which may be due to higher demands and involvement with work after hours, known as “the stress of higher status” (Schieman et al. 2006). The transition to parenthood could therefore disproportionately increase work-to-home conflict and stress of high-SES parents more than their low-SES counterparts, leading to a lower probability of having a second child. High-SES women may also be more effective at implementing lower fertility preferences through contraception after learning from negative parenting experiences (Ranjit et al. 2001).

On the other hand, a negative transition to parenthood may be more important for younger and low-SES parents in inhibiting parity progression. Taking time off work to deal with pregnancy, birth, or childcare issues may be more difficult for parents in low-skilled jobs because these jobs offer little flexibility (Anderson et al. 2003). Another reason is that even though the opportunity cost of work is, in absolute terms, lower for low-SES parents, losing a week’s salary to deal with a sick child may be more consequential for low-income parents. A third reason is differences in discounting of the future. Gregory (2007) argued that women who become parents at relatively old ages may place less value on short-term difficulties associated with becoming a parent because they are more “ready” for parenthood and have been anticipating it more intently. If high-SES parents place more weight on the future than the present, then a difficult short-term situation may be downplayed to reach the ultimate goal of a larger family.

### The German Context

Germany is the context for our study of parental well-being and parity progression. This decision is dictated by both conceptual reasons and data availability. First, understanding fertility behavior in Germany is important because the country is the largest in Europe (United Nations 2012). Second, across Europe, a major component driving the level of fertility is the transition from parity 1 to 2 (Van Bavel and Różańska-Putek 2010). In Germany, relatively low transition rates to parity 2 is an important component of persistent low fertility. The proportion of mothers who stopped at having one child has been increasing rapidly, from 25 % for the 1935–1939 birth cohort to 32 % for the 1965–1969 birth cohort (Kreyenfeld and Konietzka forthcoming). Moreover, the gap between desired fertility, which is about two, and actual fertility among Germans is very large (Bongaarts 2001), providing a fruitful ground for analyzing the determinants of the transition to parity 2.

The focus on Germany is also dictated by the fact that it is the only country with rich, nationally representative panel data available to test our hypotheses about the parental well-being and fertility behavior over a long period and for a sufficiently large sample. The German Socio-Economic Panel Study (SOEP), described in detail in the Data section, is the longest data set in the world that includes panel information about both subjective well-being and fertility behavior. Using these data, we are able to analyze how an overall measure of well-being changes annually for new parents and how these changes predict further parity progression. We measure life satisfaction before a first birth and over a long period to observe parity progression, as well as many other factors that affect progression to second birth, such as changes in partnership status and employment.

German fertility can be characterized by persistent low fertility. The period total fertility rate (TFR) has been below 1.5 since 1983. For a period of four years following the unification of East and West Germany in 1990, the TFR dropped below 1.3. There is also important regional variation. In West Germany, the TFR has been very stable, between 1.4 and 1.5, since the 1980s. However, in East Germany, the TFR declined from about 2.0 in 1980 to 1.5 in 1990, and fell below 0.8 after the unification in the early 1990s. By 2010, the TFR in East Germany climbed back to the West German level of approximately 1.4 (Goldstein and Kreyenfeld 2011; Human Fertility Database 2014).

## The Present Study

We examine whether new parents’ subjective experience of a first birth predicts whether they go on to have another child. First, we test three aspects of new parents’ trajectories of subjective well-being to see which matter most for parity progression: (1) the levels of parental life satisfaction over the transition to parenthood, (2) the gain in well-being in anticipation of a first birth, and (3) the drop in parental well-being from before to after a first birth. Based on the theories of learning and planned behavior, we hypothesize that higher levels of parental well-being over the course of transition to parenthood, greater gains in well-being leading up to birth, and smaller drops in well-being after a birth will be associated with a higher hazard of a second birth.

Second, we examine whether there are sex differences in the importance of the pattern of subjective well-being over the transition to parenthood. We hypothesize that the experiences during the transition to parenthood will be a stronger predictor of progression to a second child for women than for men because women experience the birth physically, are much more likely to take leave after the birth, and may have more power in a couple’s fertility decisions given disagreement.

Third, we examine whether parental well-being around a first birth is a stronger predictor of parity progression for highly educated parents than for those with less education, and for those with high age at first birth than for those with a lower age at first birth, net of other factors. The direction of this interaction is ambiguous, depending on whether the absolute or relative costs of temporarily dropping out of the labor force are more costly for highly or less-educated workers.

## Data

We use data from the German Socio-Economic Panel Study (SOEP), a nationally representative longitudinal study of private households run by the German Institute for Economic Research (DIW Berlin). Every year, nearly 11,000 households and more than 20,000 persons are interviewed. The data provide information on all household members, consisting of Germans living in the old (West) and new (East) German states, foreigners, and recent immigrants to Germany. The SOEP was started in 1984, with the East German states added in 1991. Wave-to-wave reinterview response rates for the SOEP have been consistently above 90 %, in line with other national panel studies such as the PSID, HRS, and BHPS (Frick 2010; Schoeni et al. 2013); and survey attrition is low (Lipps 2009).

Our analysis draws on survey waves from 1984 to 2010. Because the focus is on the parental well-being trajectory from before having children through having a birth and how that influences further parity progression, we exclude people who already had a child at first interview or who remained childless throughout the study period. We include in our analytical sample individuals whom we observe from three years before a first birth through at least two years after the first birth (*N* = 2,301). After excluding 111 respondents who reported having their first two children in one interview (twins or two singleton births within a year) and excluding another 174 respondents because of missing data on key variables (sex, age, partnership status, and educational attainment), our sample consists of 2,016 persons who experienced first births during the follow-up, 58 % of whom were observed to have a second birth over an average follow-up of 9.0 years (range 2–15) after a first birth.^1^ Although we exclude respondents who had twins at first birth, the 11 respondents who had twins after having a first child are included in the sample because they experience a second (and third) birth.

### Key Variables

The key outcome is a birth of a second child. A birth is indicated by a change in the number of biological children reported in the birth biography questionnaire. Stepchildren or adopted children are not observed in the data; thus, our analysis focuses only on biological children. This is consistent with our research goals given our interest in proceptive behavior.

Our key independent variable is parents’ subjective well-being, measured annually over the course of the transition to parenthood. Respondents were asked annually, “How satisfied are you with your life, all things considered?” Responses range from 0 (completely dissatisfied) to 10 (completely satisfied). This is a distal measure of overall positive well-being. Although this measure does not capture respondents’ overall experience of having a child, it is preferable to direct questions about childbearing because it is considered taboo for new parents to say negative things about a new child. We examine whether three aspects of respondents’ subjective well-being during the transition to parenthood are associated with a second birth, net of other important factors:

1. *Subjective well-being levels over the period of having a first child*. We measure levels of subjective well-being over the transition to parenthood, measured from two years before a child is born until the year after a first birth.
2. *Gain in well-being before first birth*. We capture the gain in well-being in anticipation of a first birth. First, we calculate a baseline level of life satisfaction for each respondent by averaging their life satisfaction level for three, four, and five years before a first birth.^2^^,^^3^ Then we sum deviations from this base level for the period two years before, one year before, and the year of first birth.
3. *Drop in well-being over the transition to parenthood*. We calculate the size of the drop in subjective well-being around a first child’s birth. We measure the difference between the maximum level of life satisfaction before a child is born (from two years before the birth through the year the child’s birth is reported) and the minimum level of life satisfaction after the birth (measured in the year the child is reported and the year after the birth is reported). This is a continuous measure that ranges from 0 if there is no drop or a gain, to 9, the maximum drop we observe in the data. This measure captures the issues raised by new parents who reported that the most common high is just before or just after the child arrives and that the most common low is during the first year after birth (Newman 2008).

### Other Variables

We test whether changes in well-being matter more for three subgroups: men versus women, younger versus older first-time parents, and more- versus less-educated parents. Age at first birth is measured as age in the first interview following the birth, and coded as less than 30 years or 30 years and older. Educational attainment at the time of first birth is also measured in the interview following the first birth and coded as less than 12 years (59 % of the sample) or 12 or more years. Our results were robust to alternative coding schemes for education. We control for variables that have been shown in previous research to also be associated with progression from parity 1 to 2. We control for country of origin with a dummy variable for whether the person was German-born (or had migrated to Germany before 1949), or immigrated to Germany in 1949 or later. A dummy variable is included for whether the respondent lived in the former East or West German states in the interview following the first birth. We also control for three time-varying variables measured annually at each interview. Partnership status measures whether the respondent was married or cohabiting, or whether the respondent was unpartnered. Household income at each interview is measured on a log scale. Labor force participation measures whether respondents were working in the last week before the survey.

## Methods

We begin with a brief overview of the characteristics of respondents at the time of their first birth, by whether they go on to have a second during the period of observation. Then, to better understand how changes in parental well-being around the first birth shape the progression to a second birth, we model the likelihood of having a second birth using event history methods. Respondents enter the model—that is, are included in the population at risk for experiencing a second birth—the year of the first birth and are censored at the second birth, exit from or end of the survey, or 15 years after the first birth, whichever comes first. We use a Cox proportional hazard model to estimate the relative hazard of a second birth. Cox regression models use the time to an event to estimate the relationship between observed covariates and the rate of occurrence of the event, taking into account that not all respondents will undergo the event and that some observations will be censored before the event occurs.

We estimate the following model:

$$ {h}_i(t)={\uplambda}_0(t) \exp \left({B}_1{W}_i+{B}_2{X}_i+{B}_3{Z}_{it}+{B}_4{R}_i+{B}_5{R}_iT+{B}_6{W}_i{X}_i+{e}_i\right), $$

where *h*_*i*_(*t*) represents the hazard, or instantaneous rate of second birth for an individual *i* at time *t*. The function λ_0_(*t*) is the nonparametric baseline hazard function, which can take any form and is the same for all individuals. The baseline hazard is shifted by the measures of parental well-being (*W*_*i*_); the time-invariant characteristics of age at first birth, gender, education at first birth (*X*_*i*_); and time-varying measures of partnership status, household income, and labor force participation (*Z*_*it*_). In testing the proportional hazards assumptions, we found that region and nativity interact with time (*R*_*i*_). Therefore, these interactions are included in the multivariate models (*R*_*i*_*T*). In the last part of the analysis, we test whether the drop in parental well-being is similarly important for men and women, by age at first birth, and by educational attainment. We test these with a series of interaction terms among sex, age at first birth or education, and measures of the experience of the transition to parenthood (*W*_*i*_*X*_*i*_). The coefficients (*B*) are estimated coefficients.

## Results

Table 1 presents sample characteristics, separating those for whom we observe a second birth and those that remain at parity 1. Life satisfaction three to five years before a first birth is high, at an average of 7.4 on a scale of 0–10. Life satisfaction increases in the year prior to and in the year of a first birth, and then decreases from the baseline level. Those who go on to have a second birth have a higher baseline life satisfaction level than those who stay at parity 1. The two groups have a similar average trajectory before a first birth, but in the year of the first birth and the year after a first birth, there are important differences. Those who have a second birth gained more in life satisfaction around the time of a first child’s birth than those who stayed at parity 1 (.20 compared with .12 units higher than baseline in the year of the first birth) and had a smaller drop in well-being in the year after the birth than the group that stays at parity 1 (−.08 compared with –.18 units lower than baseline in the year after the first birth). In other words, those who have a more difficult transition to parenthood, as measured by changes in overall life satisfaction, are less likely to have another child.

**Table 1:** Descriptive characteristics of the analytic sample, SOEP 1984–2010

	Total Analytic Sample	Observe Only First Birth	Observe Second Birth	*t* Test or χ^2^ Test^a^
	*N* = 2,016	*N* = 853	*N* = 1,163	
Parental Well-being Around First Birth				
Base life satisfaction measured 3–5 years before first birth (mean)	7.40 (1.4)	7.31 (1.4)	7.47 (1.4)	*
Life satisfaction relative to base (mean)				
2 years before first birth	−0.06 (1.5)	−0.12 (1.5)	−0.02 (1.5)	*
1 year before first birth	0.16 (1.6)	0.14 (1.5)	0.17 (1.6)	
Year of first birth	0.17 (1.6)	0.12 (1.7)	0.20 (1.6)	*
Year after first birth	−0.12 (1.7)	−0.18 (1.6)	−0.08 (1.7)	*
Total life satisfaction gain around first birth (mean)	0.27 (3.8)	0.15 (3.7)	0.35 (3.9)	*
Mean life satisfaction drop around first birth (mean)	1.41 (1.4)	1.47 (1.4)	1.38 (1.4)	*
Life satisfaction drop around first birth (categorical %)				
None	27.3	27.0	27.5	
1-unit drop	36.8	35.3	37.9	
2-unit drop	19.0	19.1	18.9	
3+−unit drop	16.9	18.6	15.6	
Other Characteristics				
Age at first birth (mean)	29.0 (5.2)	30.3 (5.9)	28.1 (4.4)	*
% Women	54.5	56.0	53.5	
Less than 12 years education at first birth	59.1	61.5	57.3	
Partnered at first birth	78.3	69.9	84.5	*
Household income (year of first birth) in Euro (mean)	41,685 (27,385)	41,310 (30,140)	41,960 (25,184)	
Working year before first birth	83.6	84.4	83.0	
Foreign-born	13.8	10.4	16.3	*
West region (%)	83.1	78.3	86.6	*
Birth year (mean)	1968 (7.1)	1968 (8.0)	1968 (6.3)	
Time to second birth (mean, median)	NA	NA	2.6, 2, (1.8)	
Time follow-up after first birth (mean, median)	9.0, 9.0, (4.6)	7.7, 6.0, (4.7)	10.0, 10.0, (4.2)	*

*Note:* Numbers: shown in parentheses are standard deviations

^a^Tests differences between those for whom only first births were observed and those who had a second birth.

*p < .05

Respondents who have a second child are also more likely to have had their first child at a younger age, to have been partnered at the time of the first child, to have been immigrants, and to have lived in West Germany than in East Germany. There are no significant differences in observed final parity by sex, educational attainment, labor force status, or household income in the year before a first birth (Table 1).

Table 2 examines whether parental well-being surrounding the transition to parenthood predicts parity progression to second birth in a multivariate hazard framework. We test three concepts with four measures of parental well-being. Model 1 tests whether life satisfaction measured annually around first birth is associated with parity progression. The variables are measured relative to the baseline level of life satisfaction, measured three to five years before a first birth. The levels of life satisfaction around first birth are not associated with parity progression: the point estimates are all close to 1 and statistically not significant both in the multivariate model and in the bivariate analysis. Next, Model 2 examines whether the gain in well-being before a first birth, in anticipation of the first birth, is associated with parity progression. We find no relationship in either the bivariate or multivariate models.

**Table 2:** Hazard ratios from Cox proportional hazard models predicting second birth, SOEP 1984–2010, *N* = 2,016

	Bivariate	Model 1	Model 2	Model 3	Model 4
Parental Well-being Around First Birth					
2 Years before first birth	1.03	1.02			
1 Year before first birth	0.95	0.97	––	––	––
Year of first birth	1.03	1.03			
Year after first birth	1.01	1.00			
Life satisfaction gain around first birth	1.01	––	1.01	––	––
Life satisfaction drop around first birth	0.95*	––	––	0.96	––
Life satisfaction drop (none)					
1-unit drop	1.03	––	––	––	0.99
2-unit drop	0.91				0.87
3+-unit drop	0.82*				0.83*
Other Characteristics					
Age at first birth	0.96***	0.94***	0.94***	0.94***	0.94***
Women (men)	0.94	0.70***	0.69***	0.71***	0.71***
12 or more years education at first birth (<12 years)	1.32***	1.65***	1.66***	1.67***	1.66***
Partnered (unpartnered)	2.27***	2.26***	2.24***	2.24***	2.25***
Household income (ln)	1.06*	1.06*	1.06*	1.06*	1.06*
Working in labor force (not working)	0.73***	0.63***	0.62***	0.62***	0.63***
German-born (immigrant)	1.20*	0.77	0.78	0.78	0.79
East region (West)	0.76**	0.56**	0.58**	0.58**	0.57**
East region × Time	––	1.14**	1.13**	1.13**	1.13**
German-born × Time	––	1.12**	1.11	1.11**	1.11*
−2 Log-Likelihood	––	−7,956.17	−8,042.09	−8,040.95	−8,039.61

*p < .05; **p < .01; ***p < .001

**Fig. 1:**
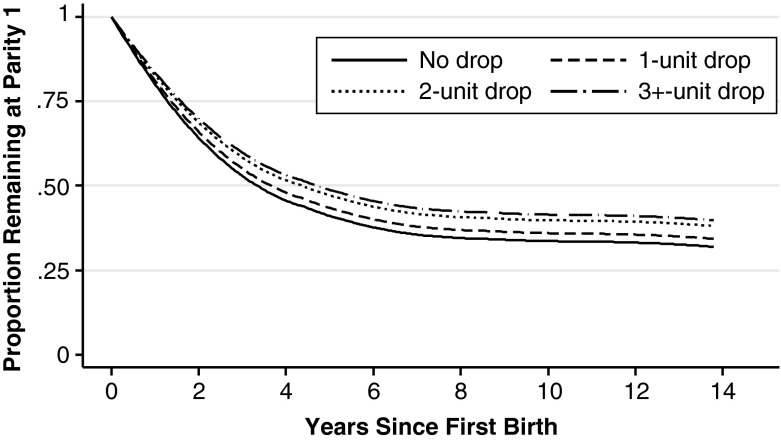
Estimated parity progression by drop in well-being at first birth, net of other factors. Estimated from Model 4 in Table 2 with other covariates held at their mean levels

Models 3 and 4 test two specifications of the drop in well-being from just before to after a first birth. The results of these models, as well as the bivariate analysis on life satisfaction drop, consistently support the hypothesis that a drop in well-being is associated with lower hazard of a second birth. Model 3 includes the drop as a continuous variable; a one-unit increase in the size of the drop is associated with a 4 % lower hazard of a second birth, and the coefficient is marginally significant (*p* < .10). To test for nonlinearity, in Model 4 we examine differences in the size of the life satisfaction drop around a first birth, comparing those with no drop, and a drop of one, two, and three or more units. The point estimates suggest that the hazard declines monotonically with the drop of life satisfaction, although only for those with a drop of three or more units in well-being around a first birth is the hazard of a second birth significantly lower (Model 4) (hazard ratio = 0.83, *p* < .05). To translate these hazard ratios into parity progression, we present in Fig. 1 the proportion who go on to have a second birth by the size of the drop in well-being at first birth, estimates from Model 4 in Table 2. The difference in the probability to have another child after five years of follow-up between those with no drop and those with a drop of three or more is about .10. For example, 60 % of those with no drop in well-being at first birth had a second birth in five years compared with 50 % of those with a large drop, net of other factors.

Because the degree to which respondents experience a drop in well-being from before to after a first birth appears to be important in predicting further parity progression, we examine the predictors of the well-being drop around first birth. Table 3 describes the characteristics of respondents who report no drop in well-being, a drop of one unit, a drop of two units, and a drop of three or more units. Overall, there are few differences between new parents who experience no drop in well-being or a drop of one. There are, however, large differences between those who experience a large drop (three or more units) and those with a small drop or no drop. Those who have a more difficult transition to parenthood are more likely to be women, have lower levels of household income, are less educated, and are less likely to be working. There are no differences by partnership status at the time of a first child, nativity, or birth cohort.

**Table 3:** Sample characteristics by size of drop in parental well-being surrounding first birth, SOEP 1984–2010, *N* = 2,016

	Drop in Well-being Surrounding First Birth			
	No Drop	Drop of 1	Drop of 2	Drop of 3+
	*N* = 550	*N* = 742	*N* = 383	*N* = 341
% Observed Second Birth	58.2	59.4	57.4	53.4
% Female	51.6	53.1	56.9	59.8
Mean Age at first birth	29.4	29.3	28.3	28.7
	(5.4)	(5.2)	(4.8)	(5.2)
% 12 or More Years Education at First Birth	41.4	45.8	35.0	36.1
% Partnered at First Birth	77.3	78.4	80.2	77.7
Mean Household Income (year before first birth)	43,706	44,763	37,884	35,998
	(27,127)	(29,902)	(22,478)	(25,771)
% Working (year before first birth)	84.5	86.9	77.8	81.2
% German-born	86.4	88.4	83.0	84.5
% West Region	82.0	83.1	84.6	83.0
Mean Birth Year	1968.8	1968.1	1968.3	1968.0
	(7.2)	(6.9)	(7.1)	(7.3)

*Note:* Numbers shown in parentheses are standard deviations.

Last, we examine whether the subjective experience of a first birth matters more or less for different subgroups: men and women, those with high and low education, and those with a high and low age at first birth. Table 3, shows that women, those with less education, and those with a lower age at first birth have larger drops in well-being around the transition to parenthood. Now we test whether the drop is equally important for these groups in predicting a second birth. Table 4 shows first that there is no difference by sex in the importance of the transition to parenthood for parity progression. Although women have larger drops in well-being over this period, the drop is equally important for men and women. Second, the drop in well-being is a significantly more important deterrent of a second birth for respondents with 12 or more years of education relative to those with less education. In the third model, we find similar results for age at first birth: a large drop in well-being around a first birth is associated with a significantly lower hazard of a second birth among those who had their first birth at age 30 or older, relative to those who became parents at younger ages.

**Table 4:** Hazard ratios from Cox proportional hazard models predicting second birth with interactions, SOEP 1984–2010, *N* = 2,016

Parental Well-being Around First Birth	Sex	Education	Age at First Birth
Women × Life Satisfaction Drop	0.95	––	––
12 or More Years Education × Life Satisfaction Drop	––	0.87**	––
Age 30 or Older at First Birth × Life Satisfaction Drop	––	––	0.90*
Life Satisfaction Drop Around First Birth	0.99	1.00	1.00
Other Characteristics			
Age at first birth	0.94***	0.94***	0.73**
Women (men)	0.75**	0.72***	0.76***
12 or more years education at first birth (<12 years)	1.67***	1.98***	1.58***
Partnered (unpartnered)	2.24***	2.24***	2.17***
Household income (ln)	1.06*	1.06*	1.04
Working in labor force (not working)	0.62***	0.62***	0.62***
German-born (immigrant)	0.78	0.77	0.82
East region (West)	0.57***	0.57**	0.59**
East region × Time	1.13**	1.13*	1.14**
German-born × Time	1.11**	1.11*	1.11**
−2 Log-Likelihood	−8,040.34	−8,036.93	−8,062.25

*Note*: Results are similar when drop in well-being is measured categorically.

*p < .05; **p < .01; ***p < .001

### Sensitivity Analysis

We conducted several robustness checks. First, we estimated results with various lengths of follow-up and found similar results when analyzing respondents for 5, 8, and 10 years after reporting a first birth. Second, we examined whether the results would change when controlling for the sex of the child: they did not. Both men and women seem to have lower drops in well-being after having a daughter rather than a son, but the sex of the first child is not associated with parity progression. Third, we ran the analysis excluding those who reported a second child in the first or second year after reporting a first child, when the drop was being measured. The sample was much smaller, and power was reduced, leading to lower levels of statistical significance, but the results yielded patterns and coefficients that were similar to those reported earlier. Fourth, we estimated models that control for period effects by using year-of-interview dummy variables. In these models, none or only one of the 21 dummy variables was statistically significant at the .05 level, so we did not include these in the final models. Last, we tested whether the results held for both the former West and East German states and found the same patterns as the results reported earlier in this article. However, the smaller samples led to less statistical power and more varied estimates.

## Discussion

A standing puzzle in demography is why fertility in many developed countries is so far below replacement level. Recent answers focus on fecundity impairments brought on by postponement of fertility, the high opportunity cost of childbearing, and partnership dissolution (Bongaarts 2001; Morgan and Rackin 2010; Quesnel-Vallée and Morgan 2003). From the purely demographic perspective, progression to parity 2 is critical because differences in fertility across developed countries are largely driven by differences in the probability of having a second child (Van Bavel and Różańska-Putek 2010). Our analysis addresses the crucial question of why those who have one do not go on to have another. We highlight how parents’ experience of a first birth shapes whether they go on to have another child. Analyzing data from Germany, a country that has had period total fertility rate below 1.5 since 1983, we find that people whose subjective well-being drops after the birth of a child are less likely to have another child than those whose subjective well-being stays at the pre-birth level. The experience during transition to parenthood is an important and understudied factor in determining completed family size. Policy-makers concerned about low fertility should pay attention to factors that influence the well-being of new parents and that might inhibit further parity progression.

Fertility research can benefit from incorporating an element of learning about parenthood. Prior research has used the macro perspective to analyze how childbearing spreads within a population (Myrskylä and Goldstein 2013); our analysis focuses on learning at the individual level. Before having children, potential parents do not know firsthand what is involved with parenting or how it will be for them. Prior research has postulated the potential importance of the psychosocial experience of parenting in fertility behavior (Newman 2008; Presser 2001). We show that the size of the drop in well-being around a first birth varies greatly; furthermore, this drop has important repercussions for completed family size, net of other factors, such as age at first birth, family resources, and partnership status. Progression to second birth within five years was 0.6 for those with no drop in well-being at first birth compared with 0.5 for those with a large drop, net of other factors.

Subjective well-being changes after a first birth may also be important for the TFR. We used parity progression rates for the 1959 birth cohort in Germany (Kreyenfeld 2002) to simulate how much the TFR would increase if those who experienced a drop in subjective well-being after the first birth, and consequently decreased parity progression, would have had the same progression to parity 2 as those who did not experience a drop in subjective well-being. The resulting increase in national-level TFR would be 0.05 children. In the German context, with TFR hovering below 1.4, this is an important change and similar in size to the effects of the recent recession on fertility (Goldstein et al. 2013).

How relevant are these findings for other low-fertility countries? In part, this depends on the size of the childless population, which dictates the proportion of the population at risk of the transition from parity 1 to 2. Childlessness among German women increased from 11 % of the 1935–1939 birth cohort to 22 % for the 1965–1969 birth cohort, the latest for which childlessness can be calculated without forecasting (Kreyenfeld and Konietzka forthcoming).^4^ Thus, approximately four-fifths of the German population will be represented by our data. This number would likely be higher in most other European countries, where the contribution of childlessness to low fertility is much lower than in Germany (Billari and Kohler 2004; Sobotka 2004, 2008). A second demographic component, which dictates how relevant this research is for other contexts, is the second birth rate. The proportion of mothers in Germany with two or more children declined from 75 % for the 1935–1939 birth cohort to 68 % for the 1965–1969 birth cohort. Relative to other European countries, second birth rates for Germany are slightly below the median. Thus, from a demographic perspective, the importance of subjective well-being in determining progression from parity 1 to 2 would be even greater in many countries than the one studied here, which has lower proportions of childless people and lower second birth rates. From a conceptual perspective, we cannot say whether subjective well-being around the transition to parenthood will be as strong a predictor of having a second child in other contexts. However, earlier qualitative research on this topic focused on different populations (e.g., U.S. in Presser 2001; Australia in Newman 2008) and came to the same conclusions as this article. Although the well-being of parents is important for the progression to a second birth, the determinants of progression to other birth orders are likely to be different, and different analysis strategies are needed to understand them (Bloom and Trussell 1984; Keizer et al. 2008; Kreyenfeld 2004; Mencarini and Tanturri 2007).

In testing for heterogeneous effects of the transition to parenthood on fertility behavior, we found that the drop in well-being around the transition to parenthood was a stronger predictor of not having a second birth for highly educated parents and those who waited longer to have a first child. We propose four possible reasons for this finding. First, highly educated parents may not enjoy the mundane tasks of parenting (Newman 2008; Presser 2001). High-SES parents may be in careers that strongly discourage childbearing (Morgan and Rackin 2010), and these parents who have a difficult transition to parenthood feel that they cannot be successful at work and have another child. Third, having a child may disproportionately increase stress of high-SES workers because of higher demands of work while at home (Schieman et al. 2006), leading to a lower likelihood of parity progression. Last, high-SES parents who learn that parenting is harder than they expected may be more prone to revise their fertility intentions downward. Alternatively, even if there were no differences in how fertility intentions are revised, high-SES parents may be better at meeting their lower fertility preferences with contraceptives than their low-SES counterparts (Ranjit et al. 2001). It is possible that the factors influencing fertility of these subpopulations are particularly important for future fertility prospects because fertility is being postponed to increasingly higher ages—for example, mean age at first birth is already above 30 in Germany (OECD 2012)—and overall educational attainment is increasing.

This analysis has limitations. First, our analysis describes the predictors and consequences of changes in overall life satisfaction for parity progression, but it cannot speak to the underlying mechanisms that determine parents’ difficulty with the transition to parenthood. These factors, such as the ease of the birth experience, level of exhaustion during the first year, and relationship stress, are not available in our survey data and are better suited to qualitative work, such as that by Newman (2008). Therefore, this research should be read alongside qualitative work. Another potential mechanism is related to “time running out.” Older parents may be influenced particularly strongly by the arrival of the first child and the experiences and responsibilities surrounding that birth because they may be aware and concerned about getting closer to the end of their reproductive age span. We are not able to examine this possibility because of data limitations. Moreover, our data do not include information on fertility desires or intentions, so we cannot analyze this. Second, we are not able to capture fertility aspirations or intention status of births in our data. Therefore, we do not know whether the drop in well-being around the first birth and the subsequent lower probability of having a second child is due to lower fertility intentions or a less positive attitude toward childbearing in the first place or whether new parents are revising their fertility intentions. However, it is also a strength to focus on transition to second birth rather than on fertility intentions or desires because it is ultimately the reproductive behavior, not intentions, that directly shape population dynamics. Nevertheless, future research on work–family conflict, planning status of births, and instrumental support would be useful to further our understanding of the specific mechanisms underlying changes in well-being around the transition to parenthood. Third, our analysis is based on annual data that do not allow analyzing changes in subjective well-being within the years before and after a birth. Prior research indicates that the risk of perinatal and postnatal depression may vary in the years before and after the birth (Banti et al. 2010), suggesting that a more nuanced analyses of the within-year changes might provide further insights into how changes in subjective well-being around the birth influence future fertility. Fourth, our measure of parental well-being is based on a distal measure of positive well-being. We capture other important factors that also matter in this analysis, but we cannot wholly exclude unmeasured factors that also contribute to changes in well-being other than having a child. Last, we examine whether the importance of changes in subjective well-being around the first birth for parity progression vary by three key demographic characteristics—sex, age, and education—but we do not analyze other potential factors. Many other factors may also be important determinants of the well-being pattern—for example, sibship size or birth order (because having had younger siblings may help anticipate the challenges of parenthood), and access to affordable childcare. Analyzing the factors determining the pattern of changes in well-being around first birth is beyond the scope of this study, but some of these factors have been analyzed in other research (Myrskylä and Margolis 2014). However, given the importance of well-being trajectories, it would be important in future studies to analyze the factors that influence the well-being trajectory around the transition to parenthood.

Despite these limitations, this article presents evidence that parents’ subjective experience of a first birth is an important and understudied factor in determining final family size. An immediate follow-up question stemming from our results is whether policy could influence the subjective well-being of new parents. In 2007, Germany implemented several new policies that were aimed at supporting new parents, and preliminary evidence suggests that these policies may have positively influenced parental well-being (Myrskylä and Margolis 2013). Further research on low fertility should address the ways in which parenting experiences throughout the life course affect fertility behavior upward or downward. After all, having children is a choice in most contexts, and adults can revise their plans as they learn firsthand about what being a parent is like.

## Footnotes

1 We cut off follow-up at 15 years after a first birth; however, results do not differ if we restrict the length of follow-up to 5, 8, or 10 years.

2 If we are missing life satisfaction for any of the periods 3, 4, or 5 years before a first birth, the baseline is calculated as the average of the nonmissing reported values.

3 Timing of the birth of a child is not known at the monthly level. We know only whether a child was born between two successive interviews that are one year apart. Years before and after the birth of a child are defined as follows. Let *x* be the first interview after a child is born. Then the interview *x* – *y* is *y* years before the child is born, and *x* + *y* is *y* years after the child is born.

4 Because of its much larger size, the West German figures are close to the German average (24 % childless for the 1965–1969 cohort). In the East, childlessness is much less common (only 17 % for the 1965–1969 birth cohort).

## References

[CR1] Ajzen I (1991). The theory of planned behavior. Organizational Behavior and Human Decision Processes.

[CR2] Ajzen, I., & Klobas, J. (2013). Fertility intentions: An approach based on the theory of planned behavior. *Demographic Research, 29*(article 8), 203–232.

[CR3] Anderson DJ, Binder M, Krause K (2003). The motherhood wage penalty revisited: Experience, heterogeneity, work effort, and work-schedule flexibility. Industrial and Labor Relations Review.

[CR4] Banti, S., Mauri, M., Oppo, A., Borri, C., Rambelli, C., Ramacciotti, D., . . . Cassano, G. B. (2010). From the third month of pregnancy to 1 year postpartum. Prevalence, incidence, recurrence, and new onset of depression. Results from the Perinatal Depression-Research & Screening Unit study. *Comprehensive Psychiatry, 52,* 343–351. 10.1016/j.comppsych.2010.08.003 21683171

[CR5] Bianchi SM, Milkie MA, Sayer LC, Robinson JP (2000). Is anyone doing the housework? Trends in the gender division of household labor. Social Forces.

[CR6] Billari FC, Kohler H-P (2004). Patterns of low and lowest-low fertility in Europe. Population Studies.

[CR7] Bloom DE, Trussell J (1984). What are the determinants of delayed childbearing and permanent childlessness in the United States?. Demography.

[CR8] Bongaarts J (2001). Fertility and reproductive preferences in post-transitional societies. Population and Development Review.

[CR9] Bongaarts J, Feeney G (1998). On the quantum and tempo of fertility. Population and Development Review.

[CR10] Brewster KL, Rindfuss RR (2000). Fertility and women’s employment in industrialized nations. Annual Review of Sociology.

[CR11] Callan VJ (1985). Choices about children.

[CR12] Cartwright A (1976). How many children?.

[CR13] Dyck I (1990). Space, time and renegotiating motherhood: An exploration of the domestic workplace. Environment and Planning D: Society and Space.

[CR14] European Commission (2006). The demographic future of Europe – From challenge to opportunity.

[CR15] Frejka T (2008). Parity distribution and completed family size in Europe: Incipient decline of the two-child family model?. Demographic Research.

[CR16] Frick, J. R. (2010). *Introduction to the Germany Socio-Economic Panel (SOEP)*. Berlin, Germany: DIW Berlin. Retrieved from http://www.diw.de/documents/dokumentenarchiv/17/diw_01.c.353304.de/soep-intro_march2010.pdf

[CR17] Goldstein JR, Kreyenfeld M (2011). Has East Germany overtaken West Germany? Recent trends in order-specific fertility. Population and Development Review.

[CR18] Goldstein JR, Kreyenfeld M, Jasilioniene A, Karaman Orsal D (2013). Fertility reactions to the “Great Recession” in Europe: Recent evidence from order-specific data. Demographic Research.

[CR19] Goldstein JR, Sobotka T, Jasilioniene A (2009). The end of “lowest-low” fertility?. Population and Development Review.

[CR20] Gregory E (2007). Ready: Why women are embracing the new later motherhood.

[CR21] Haas L (2003). Parental leave and gender equality: Lessons from the European Union. Review of Policy Research.

[CR22] Human Fertility Database. (2014). Max Planck Institute for Demographic Research (Germany) and Vienna Institute of Demography (Austria). Retrieved from http://www.humanfertility.org

[CR23] Johnson-Hanks JA, Bachrach CA, Morgan SP, Kohler HP (2011). Fertility change and variation: Understanding population trends and processes.

[CR24] Keizer R, Dykstra PA, Jansen MD (2008). Pathways into childlessness: Evidence of gendered life course dynamics. Journal of Biosocial Science.

[CR25] Kreyenfeld M (2002). Parity specific birth rates for West Germany: An attempt to combine survey data and vital statistics. Zeitschrift für Bevölkerungswissenschaft.

[CR26] Kreyenfeld M (2004). Fertility decisions in the FRG and GDR: An analysis with data from the German Fertility and Family Survey. Demographic Research.

[CR27] Kreyenfeld, M., & Konietzka, D. (Forthcoming). Childlessness in Germany. In M. Kreyenfeld & D. Konietzka (Eds.), *Childlessness in Europe: Patterns, causes and contexts*. Wiesbaden, Germany: Springer.

[CR28] Lesthaeghe, R. J., & van de Kaa, D. J. (1986). Twee demografische transities? [Two demographic transitions?]. In R. J. Lesthaeghe & D. J. van de Kaa (Eds.), *Groei of Krimp. Annual book issue of “Mens en Maatschappij”* (pp. 9–24). Deventer, The Netherlands: Van Loghum-Slaterus.

[CR29] Lipps, O. (2009). *Attrition of households and individuals in panel surveys* (SOEP Papers No. 164). Berlin, Germany: DIW Berlin. Retrieved from http://www.diw.de/documents/publikationen/73/96125/diw_sp0164.pdf

[CR30] Mencarini L, Tanturri ML (2007). High fertility or childlessness: Micro-level determinants of reproductive behaviour in Italy. Population.

[CR31] Morgan SP, Rackin H (2010). The correspondence between fertility intentions and behavior in the United States. Population and Development Review.

[CR32] Myrskylä M, Goldstein JR (2013). Probabilistic forecasting using stochastic diffusion models, with applications to cohort processes of marriage and fertility. Demography.

[CR33] Myrskylä M, Goldstein JR, Cheng YA (2013). New cohort fertility forecasts for the developed world: Rise, fall, and reversals. Population and Development Review.

[CR34] Myrskylä M, Kohler HP, Billari FC (2009). Advances in development reverse fertility declines. Nature.

[CR35] Myrskylä, M., & Margolis, R. (2013). *Parental benefits improve parental well-being: Evidence from a 2007 policy change in Germany* (MPIDR Working Paper 2013–010). Rostock, Germany: Max Planck Institute for Demographic Research.

[CR36] Myrskylä M, Margolis R (2014). Happiness: Before and after the kids. Demography.

[CR37] Newman L (2008). How parenthood experiences influence desire for more children in Australia: A qualitative study. Journal of Population Research.

[CR38] OECD. (2012). *OECD family database* [Data file]. Paris, France: OECD. Retrieved from http://www.oecd.org/social/family/database

[CR39] Presser HB, Bulatao RA, Casterline JB (2001). Comment: A gender perspective for understanding low fertility in post-transitional societies. Global fertility transition.

[CR40] Quesnel-Vallée A, Morgan SP (2003). Missing the target? Correspondence of fertility intentions and behavior in the U.S. Population Research and Policy Review.

[CR41] Ranjit N, Bankole A, Darroch JE, Singh S (2001). Contraceptive failure in the first two years of use: Differences across socioeconomic subgroups. Family Planning Perspectives.

[CR42] Rotter JB (1954). Social learning and clinical psychology.

[CR43] Schieman S, Kurashina Whitesone Y, Van Gundy K (2006). The nature of work and the stress of higher status. Journal of Health and Social Behavior.

[CR44] Schoeni RF, Stafford F, McGonagle KA, Andreski P (2013). Response rates in national panel surveys. Annals of the American Academy of Political and Social Science.

[CR45] Sobotka T (2004). Is lowest-low fertility in Europe explained by the postponement of childbearing?. Population and Development Review.

[CR46] Sobotka T, Surkyn J, Deboosere P, Van Bavel J (2008). Does persistent low fertility threaten the future of European populations?. Demographic challenges for the 21st Century: A state of the art in demography.

[CR47] Testa MR, Cavalli L, Rosina A (2011). Couple’s childbearing behavior in Italy: Which of the partners is leading it?. Vienna Yearbook of Population Research.

[CR48] Testa MR, Cavalli L, Rosina A (2014). The effect of couple disagreement about child timing intentions: A parity-specific approach. Population and Development Review.

[CR49] United Nations. (2012). *United Nations Medium Projections* [Data file]. Retrieved from http://esa.un.org/wpp/

[CR50] Van Bavel J, Różańska-Putek J (2010). Second birth rates across Europe: Interactions between women’s level of education and child care enrolment. Vienna Yearbook of Population Research.

[CR51] Wald A (1947). Foundations of a general theory of sequential decision functions. Econometrica.

[CR52] Wilson C (2004). Fertility below replacement level. Science.

